# Functional restoration of replicative senescent mesenchymal stem cells by the brown alga *Undaria pinnatifida*

**DOI:** 10.1080/19768354.2017.1292951

**Published:** 2017-03-01

**Authors:** Sin-Gu Jeong, Youn Seo Oh, I-Seul Joe, So Young Jeong, Hyo Moon Cho, Jun Sik Lee, Won Keun Oh, Tae Oh Cho, Goang-Won Cho

**Affiliations:** a Department of Biology, College of Natural Science, Chosun University, Gwangju, Korea; b Department of Life Science, BK21-Plus Research Team for Bioactive Control Technology, Chosun University, Gwangju, Korea; c Korea Bioactive Natural Material Bank, Research Institute of Pharmaceutical Science, College of Pharmacy, Seoul National University, Seoul, Korea

**Keywords:** *Undaria pinnatifida*, replicative senescence, long-term expansion, anti-oxidation, mesenchymal stromal cells

## Abstract

The brown alga *Undaria pinnatifida*, which is called Mi-Yoek in Korea, has been traditionally consumed as a health food in East Asian countries. Recent studies have reported that *U. pinnatifida* has beneficial effects on arteriosclerosis, inflammation, fat metabolism, and tumors*.* In this study, we examined the anti-senescence effects of ethanol extracts of *U. pinnatifida* (UP-Ex) in human bone marrow mesenchymal stem cells (hBM-MSCs). UP-Ex protected hBM-MSCs against oxidative injury, as determined by MTT assays. This effect was confirmed by immunoblot analysis of the oxidation-sensitive protein p53 and the apoptotic protein cleaved caspase-3. Excessive intracellular reactive oxygen species (ROS) accumulation induced by oxidative stress was moderated in UP-Ex-treated hBM-MSCs (UP-Ex-MSCs). Similarly, expression of the ROS-scavenging enzymes superoxide dismutase 1 (SOD1), SOD2, and catalase was recovered in UP-Ex-MSCs. Excessive ROS induced by long-term cell expansion (passage 17) was significantly decreased along with restoration of the senescence proteins p53, p21, and p16 in UP-Ex-MSCs. UP-Ex treatment also improved the ability of these replicative, senescent hBM-MSCs (passage 17) to differentiate into osteocytes or adipocytes, suggesting that UP-Ex ameliorates the functional decline of senescent stem cells and may provide better therapeutic efficacy in stem cell therapy.

**Abbreviations:** hBM-MSCs: human bone marrow mesenchymal stem cells; DCF: 2′,7′-dichlorodihydrofluorescein; DCFH-DA: 2′,7′-dichlorofluorescein diacetate; MTT: 3-(4,5-dimethylthiazol-2-yl-)2,5-diphenyltetrazolium bromide; PBS: phosphate-buffered saline; PFA: paraformaldehyde; RIPA: radioimmunoprecipitation assay; ROS: reactive oxygen species; SOD1: superoxide dismutase 1; SOD2: superoxide dismutase 2.

## Introduction

The steady-state level of intracellular reactive oxygen species (iROS) regulates several cellular signaling pathways, including those related to proliferation, differentiation, and inflammation, independently of cell type (Irani 2000; Hoidal 2001; Chiarugi & Buricchi 2007), while excessive ROS damage DNA, proteins, and lipids, which leads to functional decline and cellular senescence (Jung et al. 2004; Back et al. 2012; Geissler et al. 2012). Long-term *in vitro* expansion of stem cells also leads to their entering senescence with accumulation of iROS (Macip et al. 2002; Jeong & Cho 2015a), suggesting that the strict control of ROS is required to yield healthy stem cells during long-term expansion.

The antioxidant enzymes superoxide dismutase 1 (SOD1; Cu-ZnSOD), SOD2 (MnSOD), and catalase play crucial roles in the maintenance of iROS homeostasis (Johnson & Giulivi 2005). The excessive ROS production induced by oxidative stress accompanies reduction of these enzymes’ activities, and progressively induces apoptosis (Huang & Tindall 2007; Jung et al. 2015). Thus, the actions of antioxidant enzymes are closely related to ROS homeostasis and cellular protection.

The brown alga *Undaria pinnatifida* has been commercially cultivated for human consumption and used in East Asian traditional foods and healthcare diets (Murata et al. 2002; Schultz Moreira et al. 2013). Recent studies have reported that the compound fucoxanthin isolated from *U. pinnatifida* has anti-obesity effects by inducing the expression of uncoupling protein 1 in white adipose tissue (Maeda et al. 2005). Another compound, fucoidan, which was found in several species of brown algae, has a broad range of biological activities such as anti-inflammatory, antitumor, and antimetastatic activities (Kim & Lee 2012; Wang et al. 2014; Atashrazm et al. 2015). *U. pinnatifida* also contains an abundance of eicosapentaenoic acid, one of the omega-3 fatty acids associated with the prevention of inflammation, cardiovascular disorders, and mental disorders (Khan et al. 2007; van Ginneken et al. 2011).

Replicative senescence is defined as the presence of compromised DNA repair and abnormal cellular signaling, and is induced by excessive iROS. Antioxidants protect cells from these impairments and attenuate cellular senescence. In the present study, we examined the anti-senescence and anti-oxidative effects of *U. pinnatifida* ethanol extract. Our results show that this extract reduced excessive iROS accumulation and inhibited cellular senescence in human bone marrow mesenchymal stem cells (hBM-MSCs).

## Materials and methods

### Characteristics of primary hBM-MSCs and cell culture

Human BM-MSCs were purchased from CEFO (Cell Engineering for Origin, Seoul, Korea). Cells were cultured as described in our previous study (Jeong & Cho 2015a). Passage-seven (P-7) or passage-seventeen (P-17) hBM-MSCs were used for these experiments.

### Preparation of U. pinnatifida ethanol extracts (UP-Ex)

*U. pinnatifida* was harvested from the East Sea (Kang won do, Republic of Korea), washed two or three times with tap water, drained after elimination of residual salts, and dried at room temperature. The dried seaweed powder was extracted with 80% ethanol (20 times volume) for 1 week and filtered through 150-mm Qualitative Filter Paper (Hyundai Micro, Gyeonggi-do, Korea). The ethanol was evaporated using a Rotary Evaporator (Eyela, Tokyo, Japan), and the dried extract stored at −70°C for one day. After freezing, the final, dried extract was obtained by lyophilizing for 3 days in a freeze dryer (Ilshin Lab., Gyeonggi-do, Korea). The stock solutions for experimental assays were prepared by solubilizing 20 mg of UP-Ex in 1 mL of DMSO/ethanol (ratio = 1:1).

### Detection of iROS

iROS levels were measured using the cell-permeant substrate 2′,7′-dichlorofluorescein diacetate (DCFH-DA; Sigma-Aldrich, St. Louis, MO, USA), which converts to the detectable fluorescent product 2′,7′-dichlorodihydrofluorescein (DCF) inside cells. Cells were seeded in both 24-well plates (4 × 10^4^ cells/well) and 96-well plates (8 × 10^3^ cells/well) and incubated at 37°C for 24 h. Cells (80% confluence) were then treated with UP-Ex for 24 h at 37°C and incubated with 20 μM DCFH-DA at 37°C for 1 h. After washing with phosphate-buffered saline twice, the cells were incubated with hydrogen peroxide (H_2_O_2_; 1 mM) for 1 h at 37°C. Levels of iROS were measured as described in our previous study (Jeong & Cho 2015a).

### 3-(4,5-Dimethylthiazol-2-yl-)2,5-diphenyltetrazolium bromide (MTT) assay

The protective effects of UP-Ex in hBM-MSCs were evaluated by an MTT assay (Sigma-Aldrich) according to the manufacturer’s instructions. Briefly, 8 × 10^3^ hBM-MSCs were seeded onto 96-well plates. The next day, the cells were incubated with 0–10 μg/mL of UP-Ex for 24 h and then treated with 0–3 mM H_2_O_2_ for 1 h, and then analyzed by the MTT assay.

### Immunoblot analysis

Total proteins were extracted with 400 μL RIPA buffer containing 2 mM phenylmethylsulfonyl fluoride, 1 mM sodium orthovanadate, and protease inhibitor cocktail (Santa Cruz Biotechnology, Dallas, TX, USA). Immunoblot analysis was performed with specific antibodies for SOD1, catalase, p53, p21, or p16 (1:500 dilution, Santa Cruz Biotechnology, INC., Dallas, TX, USA), SOD2 (1:5000, Abcam, Cambridge, UK), cleaved caspase 3 (1:500, Merck Millipore, Darmstadt, Germany), or β-actin (1:5000, Sigma-Aldrich) for 16 h at 4°C. The appropriate horseradish peroxidase-conjugated secondary antibodies (Jackson ImmunoResearch Laboratories, West Grove, PA, USA) were used for enhanced chemiluminescence detection (GE Healthcare, Buckinghamshire, UK). The bands were quantified using the Image J software (NIH, USA).

### Osteocyte and adipocyte differentiation

The ability to differentiate into osteocytes and adipocytes was evaluated in P-17 UP-Ex-treated hBM-MSCs (UP-Ex-MSCs) according to previously described procedures (Pittenger et al. 1999; Kim et al. 2012). The efficiencies of osteogenic or adipogenic differentiation were estimated by staining with alizarin red S or with oil red O, in respectively. All stained cells were then visualized by microscopy with a Nikon Eclipse TS100 microscope. Images were captured with a Canon i-Solution IMTcam3 digital camera (Canon, Tokyo, Japan). The stained cells were quantified by the Image J software (NIH, USA) (Jensen 2013).

### Statistical analysis

All data are represented as means ± standard deviation (SD). Statistical comparisons between groups were analyzed using a paired *t* test. All *p* values < .05 were considered statistically significant.

## Results

### Protective effects of UP-Ex against oxidative stress in hBM-MSCs

**Figure 1.**
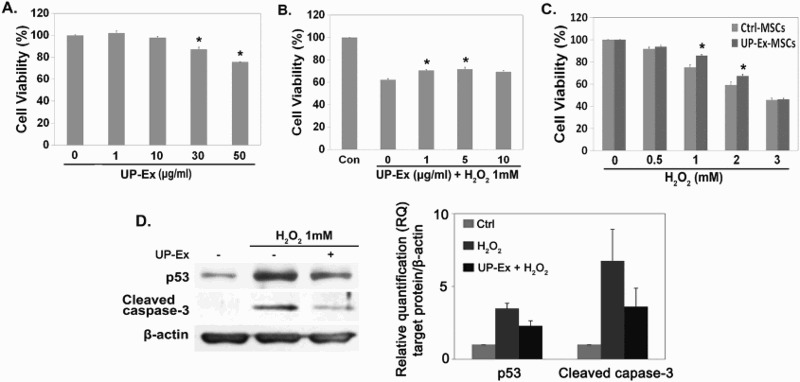
Cell-protective effects of *U. pinnatifida* extract (UP-Ex) in H_2_O_2_-treated hBM-MSCs. (a) hBM-MSCs were treated with 0–50 μg/mL UP-Ex (UP-Ex-MSCs) for 24 h and evaluated by an MTT assay. (b) hBM-MSCs were treated with 1 mM H_2_O_2_ following pre-incubation with UP-Ex (0–10 μg/mL) for 24 h and cell viability was measured by MTT assay. (c) UP-Ex-MSCs (5 μg/mL for 24 h) were exposed to 0–3 mM H_2_O_2_ for 1 h and the cell viability was evaluated by an MTT assay. Cell viability was significantly improved in UP-Ex-MSCs. (d) 5 μg/mL UP-Ex-MSCs were incubated with 1 mM H_2_O_2_ for 1 h. Total protein was examined by immunoblot analysis with antibodies against the apoptotic proteins p53, cleaved caspase-3, and β-actin. The protein expressions were quantified using the Image J software. β-actin was used as the internal standard.

To examine cytotoxicity, hBM-MSCs were treated with UP-Ex (0–50 μg/mL) for 24 h, and cell viability was measured by an MTT assay. Toxicity was not detected at ≤10 μg/mL UP-Ex treatment (Figure 1(a); *p* < .05, *n* = 4). To examine the anti-oxidative effect of UP-Ex, cells were exposed to 1 mM H_2_O_2_ for 1 h following pre-incubation with UP-Ex (0–10 μg/mL) for 24 h. The viability was significantly increased in UP-Ex-MSCs compared to untreated hBM-MSCs (Figure 1(b); *p* < .05, *n* = 3). Similarly, 5 μg/mL UP-Ex protected hBM-MSCs exposed to 1 or 2 mM H_2_O_2_ (Figure 1(c); *p* < .05, *n* = 3). To further examine these protective effects at the molecular level, apoptosis-related proteins (p53 and cleaved caspase-3) were measured by immunoblot analysis. The expression of p53 and cleaved caspase-3 was decreased in UP-Ex-MSCs compared to untreated hBM-MSCs (Figure 1(d)), which suggests that cellular damage from oxidative stress was reduced by treatment with UP-Ex.

### Restriction of excessive ROS by UP-Ex in hBM-MSCs

**Figure 2.**
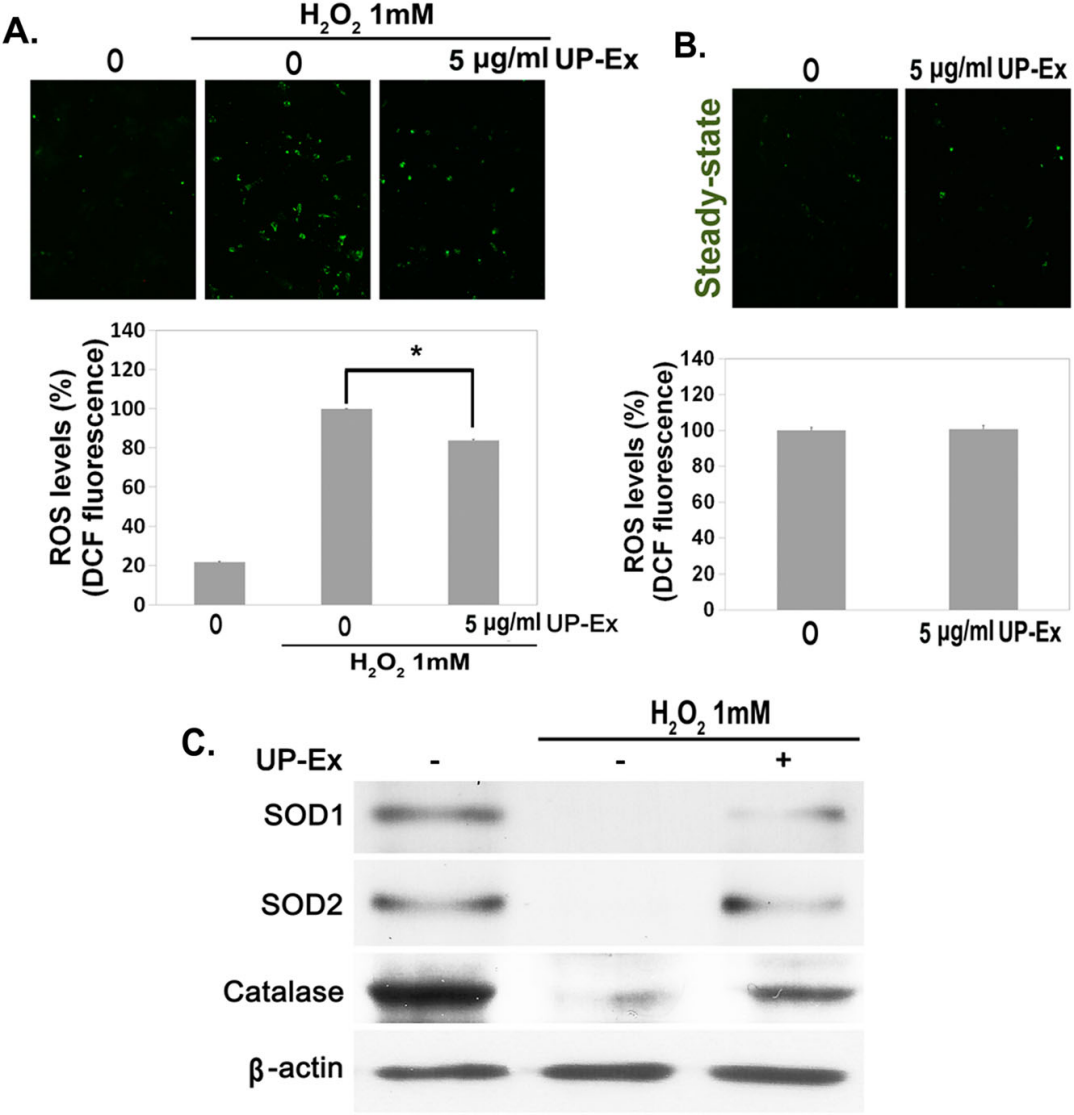
*U. pinnatifida* extract (UP-Ex) treatment reduces intracellular ROS levels in hBM-MSCs. (a) UP-Ex-treated hBM-MSCs (UP-Ex-MSCs; 5 μg/mL for 24 h) were treated with 1 mM H_2_O_2_ for 1 h, and their iROS levels were observed by fluorescence microscopy (upper panel). Fluorescence levels were quantified with a fluorescence ELISA plate reader (lower panel). (b) Steady-state iROS levels were visualized in 0–5 μg/mL UP-Ex-MSCs (upper panel) and quantified by a fluorescence-based ELISA plate reader (lower panel). (c) UP-Ex-MSCs were treated with 1 mM H_2_O_2_ for 1 h. The expression of antioxidant enzymes SOD1, SOD2, and catalase was measured by immunoblot analysis. β-actin was used as the internal standard.

Cellular damage induced by oxidative stress is caused by excessive iROS levels (Finkel & Holbrook 2000). Since UP-Ex has protective effects in H_2_O_2_-treated hBM-MSCs, iROS levels were examined in the UP-Ex-MSCs. ROS levels were significantly reduced in H_2_O_2_-treated UP-Ex-MSCs compared with untreated hBM-MSCs (Figure 2(a); *p* < .005, *n* = 4), whereas the hBM-MSCs with/without UP-Ex treatment showed no change in the steady state (Figure 2(b)). To further examine the modulation of iROS by UP-Ex, the levels of antioxidant enzymes were measured by immunoblot analysis. The expression of SOD1, SOD2, and catalase was decreased in H_2_O_2_-treated hBM-MSCs and restored by UP-Ex treatment (Figure 2(c)). These data suggest that UP-Ex reduced excessive iROS through the recovery of antioxidant enzymes, SOD1, SOD2, and catalase in hBM-MSCs.

### Recovery of antioxidant enzymes by UP-Ex in replicative senescent hBM-MSCs

**Figure 3.**
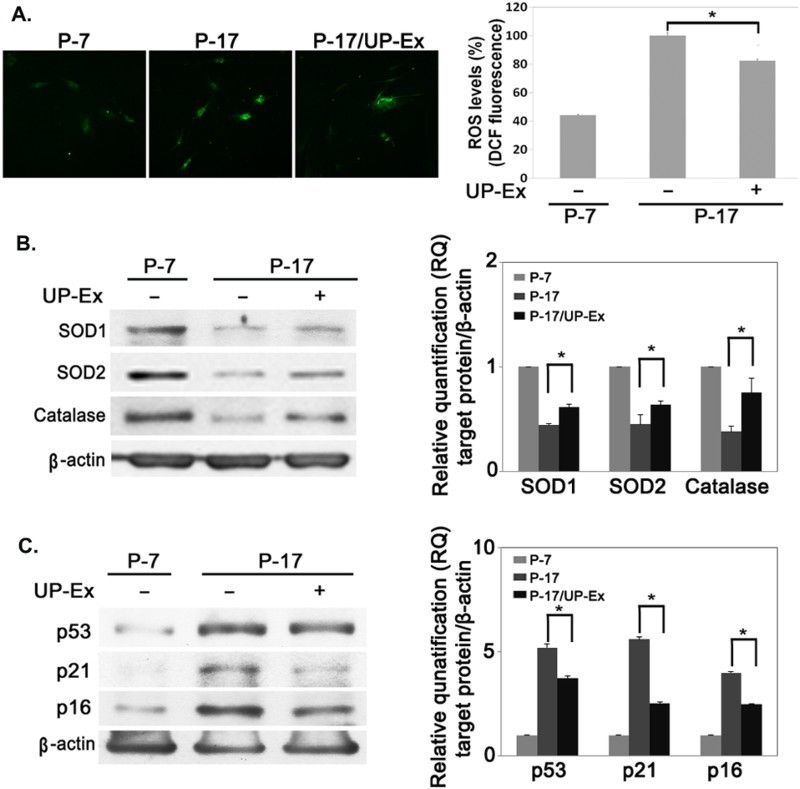
Restoration of antioxidant enzymes and reversal of senescence protein expression increases in *U. pinnatifida* extract (UP-Ex)-treated senescent hBM-MSCs. (a) Intracellular reactive oxygen species were detected with DCFH-Da in passage (P)-7, P-17, and UP-Ex-treated P-17 hBM-MSCs by fluorescence microscopy (left panel) and quantified using a fluorescence-based ELISA plate reader (right panel). (b) Total protein from P-7, P-17, and UP-Ex-treated P-17 hBM-MSCs were examined by immunoblot analysis with antibodies against SOD1, SOD2, catalase, and β-actin. (c) The expression of the senescence proteins p53, p21, and p16 was examined by immunoblot analysis. The bands in (b) and (c) were quantified by the Image J software and normalized with β-actin.

Our previous study showed that stem cells made senescent by long-term expansion accumulate iROS and exhibit reduced antioxidant enzyme expression (Jeong & Cho 2015a). To test whether the excessive ROS induced by long-term culture is reduced by UP-Ex treatment, hBM-MSCs were expanded up to P-17 and stained for the presence of iROS. The increased ROS in P-17 cells was significantly decreased in P-17 UP-Ex-MSCs (Figure 3(a); *p* < .05, *n* = 4). Immunoblot analysis of antioxidant enzymes showed that the expression of SOD1, SOD2, and catalase was decreased in P-17 cells and restored in P-17 UP-Ex-MSCs (Figure 3(b); *p* < .05, *n* = 3). In addition, the senescence proteins p53, p21, and p16 were increased in P-17 cells and this increase was reversed in P-17 UP-Ex-MSCs (Figure 3(c); *p* < .05, *n* = 3). These data suggest that UP-Ex reverses cellular senescence through recovery of antioxidant enzymes.

### Restoration of differentiation capacity by UP-Ex treatment

**Figure 4.**
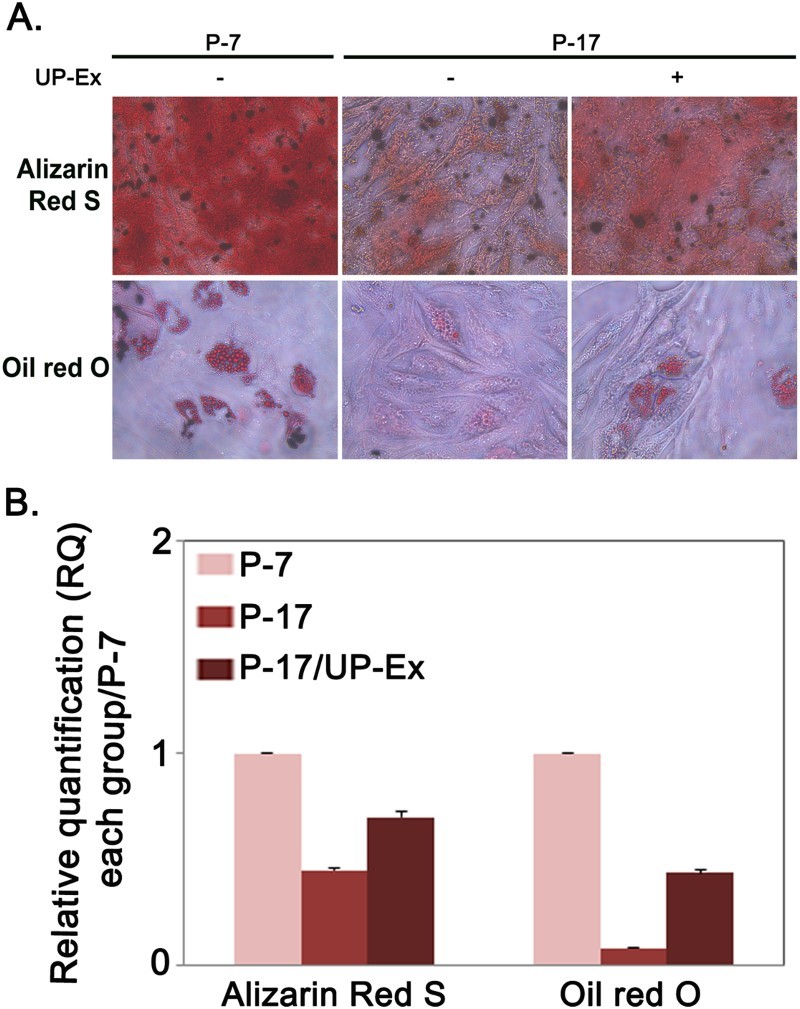
Improvement of differentiation potential in *U. pinnatifida* extract (UP-Ex)-treated senescent hBM-MSCs. (a) hBM-MSCs at passage (P)-7 or P-17, with or without UP-Ex treatment at P-17, were induced to differentiate into osteocytes (upper panels) and adipocytes (lower panels). Differentiation capacities were evaluated by alizarin red S or oil red O staining. (b) The Alizarin red S or oil red O stained cells were quantified using the Image J software.

If UP-Ex has anti-senescence effects in replicative senescent stem cells, cells treated with UP-Ex may recover their differentiation potential. To confirm this hypothesis, long-term (P-17) cultured hBM-MSCs were treated with 5 μg/mL UP-Ex for 24 h and differentiated into osteocytes and adipocytes. P-17 hBM-MSCs exhibited reduced differentiation potential compared to P-7 cells, and UP-Ex treatment of P-17 cells restored this potential compared to untreated P-17 hBM-MSCs (Figure 4). These results suggest that the decline in differentiation capacity caused by cellular senescence was at least partially reversed by UP-Ex treatment.

## Discussion

Several useful compounds such as fucoxanthin and fucoidan have been isolated from *U. pinnatifida* (Deux et al. 2002; Maeda et al. 2005). Major benefits of these compounds have been demonstrated in arteriosclerosis, inflammation, fat metabolism, and antitumor therapy (Kim & Lee 2012; Roux et al. 2012; Kim, Jeon, et al. 2014; Atashrazm et al. 2015). In this study, to demonstrate the antioxidant and anti-senescence properties of *U. pinnatifida*, UP-Ex was used to treat hBM-MSCs, which were then evaluated for protection against senescence and oxidation. Treatment with UP-Ex resulted in enhanced protection against oxidative stress in hBM-MSCs (Figure 1). The oxidation-sensitive protein p53 was increased upon H_2_O_2_ treatment, and this increase was partially reversed when hBM-MSCs were pretreated with UP-Ex. Similarly, expression of the apoptotic protein cleaved caspase-3 was reduced in UP-Ex-MSCs (Figure 1(d)).

Antioxidant enzymes such as SOD1, SOD2, and catalase are known as major scavengers of iROS (Johnson & Giulivi 2005). Expression of these enzymes is decreased in cells exposed to excessive oxidative stress, resulting in the accumulation of iROS (Huang & Tindall 2007; Jeong & Cho 2015b). Since we have shown that UP-Ex pretreatment protects against oxidative stress in hBM-MSCs, it is reasonable to assume that this protection is due to moderated iROS levels. To address this hypothesis, ROS levels were measured in H_2_O_2_-treated MSCs following pre-incubation with UP-Ex. As expected, the increased ROS levels were moderated in UP-Ex-MSCs (Figure 2(a) and (b)), which was likely caused by restoration of the ROS-scavenging enzymes SOD1, SOD2, and catalase (Figure 2(c)).

Adult stem cells have been used for stem cell therapy in degenerative diseases (Barry & Murphy 2013; Farini et al. 2014; Kim, Kim, et al. 2014). However, previous studies have shown that stem cells gradually display a senescent phenotype during long-term expansion followed by accumulation of iROS (Oka et al. 2015; Su et al. 2015; Jeong & Cho 2015a). Nevertheless, cell expansion is required to obtain sufficient amounts of donor stem cells for stem cell therapy (Wagner & Ho 2007), which is a dilemma that needs to be resolved to obtain healthy donor stem cells. Our previous study has shown that elevation of ROS levels plays important role in the progression of cellular senescence. Treatment of antioxidant ascorbic acid reduced intracellular ROS and improved the differentiation capacity in senescent stem cells (Jeong & Cho 2015a). In this study, we showed that excessive iROS induced by long-term cell expansion was significantly decreased in UP-Ex-pretreated MSCs (Figure 3(a) and (b)), which accompanied a significant reversal of the senescence-related proteins p53, p21, and p16 (Figure 3(c)), suggesting that cellular senescence induced by cell expansion may be partly ameliorated by pre-incubation with UP-Ex.

If UP-Ex treatment downregulates excessive iROS accumulation and reduces the expression of senescence-related proteins in senescent stem cells (P-17), it may also improve the differentiation potential of stem cells. In support of this hypothesis, long-term cultured hBM-MSCs (P-17) pretreated with UP-Ex could be differentiated into osteocytes or adipocytes, confirming the differentiation potential of UP-Ex treated P-17 MSCs (Figure 4). This result suggests that treatment with UP-Ex can recover the functional decline that is characteristic of replicative senescent stem cells.

In conclusion, we have shown that an ethanol extract of *U. pinnatifida* moderates excessive iROS and reverses replicative senescence with an increase in antioxidant enzymes in hBM-MSCs. This study has also shown that UP-Ex treatment restored the differentiation potential of replicative senescent (P-17) hBM-MSCs, suggesting that UP-Ex ameliorates the functional decline of senescent stem cells. Combined treatment with UP-Ex may provide better therapeutic efficacy in stem cell therapy.
